# Examining dynamic changes in prefrontal connectivity and internalizing symptoms during the first and third trimesters of pregnancy

**DOI:** 10.1111/jne.70252

**Published:** 2026-09-04

**Authors:** Emily B. K. Thomas, Micah A. Williams, Patrick Breheny, Abigél Miskolczi, Ö. Ece Demir‐Lira

**Affiliations:** ^1^ Department of Psychological and Brain Sciences University of Iowa Iowa City Iowa USA; ^2^ Department of Biostatistics University of Iowa Iowa City Iowa USA

**Keywords:** distress, internalizing symptoms, longitudinal, prefrontal connectivity, pregnancy

## Abstract

Pregnancy is characterized by pronounced neuroplasticity and heightened risk for psychopathology. While previous research has primarily examined postpartum depression, less work has investigated changes in internalizing symptoms measured dimensionally across the peripartum period. Furthermore, limited research has identified functional changes in the prefrontal cortex (PFC) across pregnancy, a brain area implicated in psychopathology. Understanding the dynamic changes in internalizing symptoms and PFC neuroconnectivity throughout pregnancy and their interrelations is crucial for early identification of perinatal psychopathology. This pre‐registered longitudinal study examined changes in and associations between internalizing symptoms and PFC functional connectivity throughout the first and third trimesters of pregnancy. Pregnant individuals recruited from a community sample of females engaged in prenatal care (*N* = 60, *M*_age_ = 32.31, SD = 5.34, 90% White, 91.7% non‐Hispanic/Latine) completed structured clinical interviews assessing mood and anxiety symptoms and resting‐state functional near‐infrared spectroscopy measuring PFC connectivity during the first and third trimesters of pregnancy. From the first to third trimester, longitudinal results revealed a significant decrease in symptoms of distress and fear and significant increases in neuroconnectivity anchored in left medial frontal areas of the PFC. Cross‐sectionally, higher first trimester fear was associated with lower within‐region right medial PFC and higher within‐region left medial PFC connectivity. Longitudinal decreases in distress from first to third trimester were associated with increased connectivity between left medial and right lateral PFC, alongside weakened connectivity between bilateral medial frontal regions. These findings demonstrate that internalizing symptoms relate to prefrontal connectivity from first to third trimester in a community sample of pregnant women. Examining internalizing symptoms dimensionally across pregnancy allows for characterization of the full range of symptoms that impact pregnant individuals. Future research should explore prevention and intervention implications of the relations between neural profiles and internalizing symptoms during pregnancy to improve long‐term outcomes for the mother and child.

## 1 INTRODUCTION

Pregnancy is a period characterized by biopsychosocial change. For the birthing parent, this period is also characterized by elevated risk for psychopathology. Identifying the psychiatric changes that occur during this period, as well as the adverse outcomes (e.g., disrupted mother–infant bonding, long‐term psychiatric symptoms) that are associated with psychiatric symptoms, is critical to facilitate prevention and intervention efforts. As perinatal psychopathology impacts not only the birthing parent, but also the developing infant and the family unit, it is imperative that early identification and intervention are prioritized. Perinatal psychopathology research has primarily focused on measurement and treatment of postpartum depression, which is prevalent and consequential, affecting an estimated 10%–18% of individuals following childbirth.^1^, ^2^ Given evidence supporting heterogenous trajectories of the development, severity, and duration of perinatal depression,^3^ considering early identification of vulnerability and symptoms during the course of pregnancy is critical.

Depressive and anxiety disorders are prevalent, impairing, and consequential during the perinatal period.^2^ At the first, second, and third trimesters of pregnancy, previous research has estimated prevalences of depressive symptoms to range from 7% to 13% and to peak during the postpartum period at 18%.^1^, ^4^ Similarly, a meta‐analysis found the prevalence of anxiety symptoms to be 18.2%, 19.1%, and 24.6% during the first, second, and third trimesters, and 17.8% at 1‐month postpartum.^5^ Perinatal depression and anxiety were associated with adverse outcomes for both mother and infant, including psychosocial difficulties, birth complications, infant attachment problems, maltreatment, and more.^6^ Screening for depressive and anxiety symptoms during pregnancy and postpartum is recommended, but screening measures are necessarily short and limited in scope.^7^ Additionally, these screening measures focus on the *Diagnostic and Statistical Manual of Mental Disorders, Fifth Edition, Text Revision* (DSM‐5‐TR)^8^ diagnoses when evidence suggests significant overlap between symptom domains and comorbidities throughout the perinatal period that align better with the well‐studied hierarchical taxonomy of psychopathology.^9^, ^10^ Dimensional measurement of internalizing psychopathology allows for symptoms to cross diagnostic categories and focuses on severity of symptoms across domains rather than categorical diagnosis.

Our main goal in the current study is to examine longitudinal changes in internalizing psychopathology during pregnancy and pinpoint the neural basis of these changes. Next, we review relevant literature on neural changes associated with pregnancy, and then the literature linking these neural changes to psychopathology during pregnancy and the postpartum time window. To provide a comprehensive background, we further provide details on neuroimaging modalities during rest and task states in relation to pregnancy and mental health.

### 1.1 Neural changes in the pregnancy and postpartum period

Pregnancy is a period of neuroplasticity associated with pronounced structural and functional changes across the brain. In terms of structural changes, longitudinal studies leveraging magnetic resonance imaging have documented decreases in gray matter volume, cortical thickness, and surface area from pre‐ to post‐pregnancy, and these changes were absent in nulliparous control women (non‐mothers) observed over the same time period.^11^, ^12^, ^13^, ^14^, ^15^ More recent studies have reported a U‐shaped trajectory in gray matter volume that partially recovers during postpartum years.^15^ Increases in white matter microstructure integrity throughout pregnancy^14^ and increased white matter volume have also been reported in first‐time mothers compared to nulliparous women in the perinatal period.^16^

Some of the networks that show prominent changes throughout pregnancy are the attention and sensory networks, driven particularly by network‐level changes in the dorsolateral prefrontal cortex (PFC), inferior parietal lobe, postcentral gyri, posterior cingulate, and somatosensory areas.^11^, ^12^, ^13^, ^14^ The frontoparietal and default‐mode networks (DMN), involved in higher‐order executive function and internal awareness, neural basis of self, and self‐referential processing, also undergo significant transformation.^11^, ^14^, ^17^, ^18^ More broadly, pregnancy is associated with changes in areas that facilitate reward, salience, emotion regulation, and social cognition during the transition to motherhood.^17^ Together, these neural changes are thought to contribute to the fine‐tuning process that renders the maternal brain more flexible, efficient, and responsive during caregiving.

#### 1.1.1 Prefrontal cortex during pregnancy and postpartum period

Among the wide array of regions that undergo transformation during pregnancy and postpartum, the PFC plays a particularly important role. The PFC is central to higher‐order processing, such as cognitive control, executive function, and emotion regulation,^19^ in part through its regulation of limbic and subcortical structures such as the amygdala. It supports a wide array of regulatory and goal‐directed actions spanning mental, internal, emotional, and motor behaviors.^19^ Although its subdivisions vary, the PFC is most commonly divided into dorsolateral (dlPFC), dorsomedial (dmPFC), ventromedial (vmPFC), and orbital prefrontal regions, each linked to distinct regulatory functions. The dorsolateral and frontopolar regions are particularly important for emotion regulation, inhibitory processes, integrative processes, and control of information, as they receive processed sensory information from posterior association cortices. The orbitofrontal region is considered key for emotion regulation. The medial PFC, a main hub of the DMN, is involved in self‐referential thinking, as well as theory of mind, and cognitive empathy. The lateral PFC is involved in regulating one's own emotions via processes such as verbal regulation or suppression of affective content.^20^ In terms of Brodmann areas (BA), the PFC consists of BA8 to 14 and BA44 to 47.^21^ Together, these functions position the PFC as central to the regulatory demands of the transition to motherhood.

Relatedly, both animal and human studies have highlighted significant changes and group differences in PFC throughout pregnancy and the perinatal period, with some relating these changes to variability in mental health and examining how such changes relate to maternal caregiving behaviors.^22^, ^23^, ^24^ Changes in PFC are among the most consistently reported findings.^11^, ^13^ PFC changes have been examined using both task and resting‐state paradigms across cross‐sectional and longitudinal designs. Among functional neuroimaging studies, task‐based paradigms have primarily used functional magnetic resonance imaging (fMRI) and focused on the postpartum period. For example, new mothers have shown differing PFC activation patterns when viewing infant faces compared to nulliparous women.^11^ Furthermore, when mothers viewed pictures of their own infants, the medial PFC and inferior frontal gyri, the PFC regions which exhibited the most structural change, were most strongly activated.^11^ Although prior work has uniquely focused on functional differences in the postpartum period,^11^, ^16^ research examining these changes during the gestational period is growing. To promote safety and comfort, an increasing number of task‐based studies have leveraged a non‐invasive imaging method, functional near‐infrared spectroscopy (fNIRS), to track changes in pregnancy and the perinatal period. Prior task‐based studies using fNIRS have shown that pregnant women show significant PFC activation to fear‐stimuli compared to rest.^25^ Notably, increased left PFC and decreased medial PFC activation have been associated with higher distress.^25^ Others have shown that in the PFC, especially the dlPFC and vmPFC, responsivity to infant cries and faces changes throughout gestation.^26^ Another study showed increased activation in right PFC for mothers when differentiating facial emotions, an effect not observed in non‐mothers.^27^ Beyond task‐related responses, resting‐state paradigms measure intrinsic functional activity during quiet wakefulness in the absence of cognitive, sensory, or social stimulation.

Importantly for our purposes, resting‐state paradigms are particularly powerful in revealing functional patterns associated with pregnancy.^28^ Indeed, resting‐state fMRI studies have shown increased global and local network efficiency in prefrontal areas in mothers compared to nulliparous women in the postpartum period,^29^ increased temporal coherence of prefrontal areas through pre‐conception, late pregnancy, and postpartum,^12^ as well as longitudinal changes in connectivity of prefrontal regions to other affective regions, such as the amygdala, in postpartum mothers compared to controls.^18^ One recent fMRI study collected dense‐sampling resting‐state data from one individual who underwent in vitro fertilization, and 26 scans were completed from pre‐conception through the postpartum period.^14^ Another longitudinal electroencephalogram (EEG) study showed increased functional coupling or coherence of prefrontal areas in the postpartum time window compared to pregnancy.^30^

Taken together, very little is known about the longitudinal changes in resting‐state connectivity spanning the pregnancy period. Recent work emphasizes the need for dense‐sampling studies (small *n*, large *p* designs) to capture the changes in the brain with higher temporal resolution for more extended periods of time.^31^ Particularly, prefrontal neuroconnectivity changes during pregnancy and predictors of diverging neural trajectories remain understudied, motivating the primary aim of the current study.

### 1.2 Prefrontal cortex and psychological symptoms

Changes in mental health may contribute to the diverging neural trajectories observed during pregnancy. The PFC has been implicated in a variety of psychological symptoms, including depression and anxiety. Resting‐state fMRI studies report alterations in functional connectivity of PFC, including reduced connectivity in various regions of PFC in patients with depression compared to healthy groups,^32^, ^33^ with pronounced differences in vmPFC and dlPFC connectivity.^34^ Recent fNIRS studies distinguished between individuals with severe mental illness and healthy controls where PFC connectivity was reduced among individuals with affective disorders when compared to healthy controls.^35^ Given that fNIRS was used to successfully distinguish healthy individuals from those with psychiatric disorders, these findings highlight the potential utility of fNIRS as a candidate biomarker.^35^ Overall, PFC function seems to correlate with psychological symptoms.

With respect to the relation between psychological symptoms and neural changes during pregnancy and the perinatal period, one task‐based fNIRS study showed that increased PFC activation during processing of fear‐relevant stimuli was associated with higher general distress and anxiety in pregnant women.^25^ In terms of resting‐state measures, mothers with postpartum depression showed altered connectivity in prefrontal networks, such as in the dorsomedial PFC.^25^ For example, one resting‐state fMRI study showed that mothers with postpartum depression exhibit increased homogeneity, a measure of local synchronization of spontaneous activity, in medial frontal areas, but reduced synchronization in temporal areas.^36^ Women with postpartum depression showed greater connectivity of dmPFC with the rest of the DMN compared to controls, and the degree of connectivity was positively correlated with depression scores.^37^ Another study using resting‐state fNIRS showed decreased network connectivity in the right inferior frontal gyrus and broader PFC.^38^ Taken together, although an emerging body of work links resting‐state neuroconnectivity patterns to psychological symptoms especially in the postpartum period, to our knowledge, how dynamic changes in psychological symptoms during pregnancy relate to neural connectivity changes remains uncharted.

### 1.3 Objectives of the present study

The prior literature suggests that pregnancy and the postpartum period are a period of pronounced structural and functional change in prefrontal areas and that these changes are linked to psychological symptoms. However, how variability in psychological symptoms throughout pregnancy relates to variability in prefrontal trajectories remains unknown. The present study sought to determine the dynamic relation between internalizing symptoms and neuroconnectivity throughout the first and third trimesters of pregnancy among a community sample of individuals receiving prenatal care. Historically, this time period, also referred to as a gestational blind spot in neuroimaging research, has not been the focus of functional imaging studies due to comfort concerns and sensitivity to movement. fNIRS is a safe, non‐invasive, portable, and ecologically valid measure of neuroconnectivity and presents a reasonable alternative to costly and time‐intensive methods. Using a structured interview measuring internalizing psychopathology dimensionally, alongside resting‐state fNIRS, the authors utilized a longitudinal study design to clarify this relation.

The primary aims of this study were to: (1) examine changes in dimensions of internalizing psychopathology (distress, fear, obsessive‐ compulsive disorder (OCD)/mania) from the first to third trimester of pregnancy; (2) examine changes in functional connectivity in the PFC during resting state from first to third trimester of pregnancy; and (3) examine how changes in internalizing psychopathology and changes in functional connectivity are related from the first to third trimesters of pregnancy. The authors hypothesized that internalizing psychopathology symptoms would decrease from first to third trimester. Based on prior work comparing pre and post‐pregnancy, we hypothesized that we would observe increased connectivity in the PFC from first to third trimester.^12^, ^29^ Lastly, we suspected that stable or decreasing symptoms of psychopathology would be related to increased PFC connectivity between first and third trimesters.^35^ This study and the relevant hypotheses were pre‐registered on Open Science Framework (OSF) (https://osf.io/4sy95/overview?view_only=a2b174354ad9489ea7881225b3441a23).

## 2 METHOD

### 2.1 Procedures

Ethical approval was granted by the University of Iowa institutional review board (IRB) (Protocol #: 202106060). Participants were recruited via three avenues. University mass emails (reaching approximately 40,000 individuals) were disseminated approximately monthly, and flyers and brochures were placed in medical settings and local businesses. An existent registry of pregnant individuals at the university hospital was utilized to recruit potentially eligible pregnant individuals. Those who appeared eligible following completion of a Research Electronic Data Capture (REDCap) screening survey were contacted by phone to review consent information and study procedures. There was no inclusion or exclusion criterion related to presence or absence of psychiatric conditions, with the aim of recruiting a community sample of individuals utilizing prenatal care. The potential participants had until the end of the 14th week of pregnancy to decide whether to participate. Consent for the study procedures included survey and interview completion, and neuroimaging sessions were optional. Storage of contact information for future studies was optional. Following verbal consent discussion, an electronic consent form was completed via REDCap's e‐consent feature. Participants consented for self and their infant.

Following electronic written consent via REDCap, participants were provided the baseline survey via REDCap. The structured interview occurred via phone or in‐person, based upon participant preference. In its validation study, assessment mode via phone or in‐person had no detectable effect on the interview's factor structure, suggesting delivery method has little impact on scores.^39^ The interview was scheduled as close in time to survey completion as possible. The fNIRS session was scheduled following survey completion and was often paired with in‐person interviews, but when the interview occurred by phone, the fNIRS session was scheduled as close in time as possible to the interview and survey completion date. The survey and interview each took approximately 1‐h to complete, and the fNIRS sessions took approximately 30 min to complete. fNIRS recordings took place in a quiet, dimly‐lit room. Participants were shown a fixation cross on a black background on a 22‐in. monitor and asked to relax themself, their mind, and to look at the cross on the screen for a recording of 7 min. Participants were contacted for a similar sequence (survey, interview, and fNIRS) during the third trimester, ideally all occurring between 30 and 32 weeks gestation, but contingent upon participant availability. Further follow‐up assessment occurred in the postpartum period as detailed in the pre‐registration, but only pregnancy data are presented herein.

Interviews were audio‐recorded and stored on the university secure drive. Audio recordings were utilized to verify scoring by a second rater. fNIRS sessions were video and audio‐recorded so that changes in neural activity could be linked to any in‐session occurrences or abnormalities (e.g., loud unexpected noise).

Participants were compensated for participation using a gift card in the following amounts—survey: $20; interview: $25; and fNIRS: $25. Participants could opt out of any of the procedures or questions that they did not wish to complete.

### 2.2 Participants

Eligible pregnant individuals were aged 18–55, were receiving prenatal care at the local academic medical center, the University of Iowa Hospitals and Clinics, and were recruited prior to 15 weeks' gestation. Exclusion criteria were non‐singleton pregnancy, inability to read/speak English fluently, and traumatic brain injury history. Non‐singleton pregnancies were excluded due to an infant and parent–child assessment included in the postpartum period that necessitates a single infant. Primiparous and multiparous participants were included; there was no exclusion criterion based on the number of prior live births. Participants provided electronic written informed consent via secure REDCap e‐consent for self and infant. The fNIRS portion of the study was optional, and participants could decline any aspect of the study or question that they did not wish to answer. Participants were included in all analyses for the completed assessments, with 60 individuals completing at least one assessment at each time point and thus eligible for inclusion. For inclusion in the change analyses between connectivity and internalizing symptoms, individuals needed to complete the first and third trimester structured interviews and fNIRS assessments (*n* = 42). Of the remaining 18 individuals, 11 completed only first trimester assessments, three only completed the interview at each time point, one completed both interviews but only one fNIRS, one completed only third trimester assessments, and two completed both interviews but declined the third trimester fNIRS. Demographic information was obtained from the first trimester survey.

The sample consisted of 60 pregnant participants ranging from ages 19.94 to 46.43 years (*M* = 32.31, standard deviation [SD] = 5.34), and the majority identified as White race (90%), non‐Hispanic/Latine (91.7%), and married (88.3%). The sample was highly educated, with 95% having some college or more education. The majority of the sample reported family income of >$80,001 (65%). For more information, see Table 1.

**TABLE 1:** Descriptive characteristics of the sample.

Sample characteristics (*N* = 60)	*N* (%)
Age, *M* (SD, range)	32.31 (5.34, 19.94–46.43)
Race	
African American/African/Black	0 (0.0%)
Asian/Indian	1 (1.7%)
Caucasian	54 (90.0%)
Native American/Alaska Native	0 (0.0%)
Pacific Islander	0 (0.0%)
Multiple	3 (5.0%)
Other	2 (3.3%)
Ethnicity	
Hispanic/Latine	5 (8.3%)
Non‐Hispanic/Latine	55 (91.7%)
Years education, *M* (SD)	18.53 (2.97)
Education	
Doctoral degree (PhD, MD, JD, EdD, etc.)	10 (16.7%)
Master's degree	16 (26.7%)
Some graduate school	4 (6.7%)
Bachelor's degree	21 (35.0%)
Associate's degree	3 (5.0%)
Some college	3 (5.0%)
Completed trade school	0 (0.0%)
High school graduate/GED	3 (5.0%)
Did not finish high school	0 (0.0%)
Family income	
$80,001 or greater	39 (65.0%)
$60,001–$80,000	7 (11.7%)
$40,001–$60,000	6 (10.0%)
$20,001–$40,000	6 (10.0%)
Up to $20,000	0 (0.0%)
Missing	2 (3.3%)
Marital status	
Married	53 (88.3%)
Living with a partner	7 (11.7%)
Committed relationship living separately	0 (0.0%)
Divorced	0 (0.0%)
Separated	0 (0.0%)
Single	0 (0.0%)
Conception method	
Natural	53 (88.3%)
IUI (intrauterine insemination)	2 (3.3%)
IVF (in vitro fertilization)	4 (6.7%)
ICSI (intracytoplasmic sperm injection)	1 (1.7%)
Gestational week: first trimester, *M* (SD, range)	10.45 (2.66, 5–15)
Gestational week: third trimester, *M* (SD, range)	29.43 (1.07, 28–33)

*Note*: Gestational week is reported for time that the survey was completed, when the data collection begins for that visit (survey, IMAS‐R, fNIRS).

Abbreviation: SD, standard deviation.

### 2.3 Measures

#### 2.3.1 Internalizing symptoms

The Interview for Mood and Anxiety Symptoms‐Revised (IMAS‐R) is a structured interview that assesses a broad range of mood and anxiety symptoms and is scored dimensionally, aligning with hierarchical conceptualizations of psychopathology.^10^, ^39^, ^40^ Each item of the interview is scored on a three‐point rating scale: absent (0), subthreshold (1), or above‐threshold (2). The interview assesses all Diagnostic and Statistical Manual of Mental Disorders, Fourth Edition (DSM‐IV) and International Classification of Diseases, Tenth Revision (ICD‐10) emotional disorder symptoms, as well as 31 subscales reflecting components of emotional disorder symptoms (e.g., dysphoria, anhedonia, lassitude, suicidality, agitation, retardation, appetite loss, insomnia, intrusions, and avoidance). Scores across these subscales may be totaled to produce a single internalizing factor score. Additionally, the IMAS‐R's dimensional approach results in three broad domains of internalizing symptoms: distress, fear, and OCD/mania. The distress domain includes symptoms of dysphoria, anhedonia, lassitude, avoidance, irritability, and generalized anxiety, among others, and scores range between 0 and 124. The fear domain includes social anxiety, agoraphobia, and specific phobia symptoms and has a score range of 0–62. The OCD/mania domain includes obsessions, compulsions, euphoria, and hyperactive cognition symptoms, with scores ranging from 0 to 70. These domains are distinct and scored separately, meaning one may score high in one domain but low in the others. The dimensional approach to symptoms looks beyond diagnostic categories and instead focuses on symptom severity across categories, offering an alternative to diagnoses that are often co‐occurring and highly correlated (e.g., depression and generalized anxiety symptoms fall into the distress domain). Within our sample, reliability across IMAS‐R domains was adequate at both the first trimester (distress *α* = .94; fear *α* = .92; OCD/mania *α* = .88) and third trimester (distress *α* = .92; fear *α* = .87; OCD/mania *α* = .75). To the best of the authors' knowledge, the IMAS‐R has primarily been investigated in research contexts.

#### 2.3.2 fNIRS measures

##### fNIRS data acquisition

**FIGURE 1:**
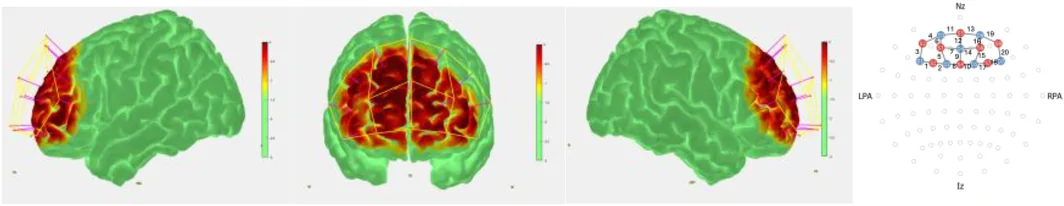
First three panels (left, front, and right views) represent the photon sensitivity profile for a representative subject scan. Channels are formed between adjacent source‐detector pairs and represented with yellow lines and labeled accordingly. Sensitivity profile for photon propagation depth is presented as a heat map in log10 (mm^−1^). units. The fourth panel represents the location of light sources and detectors are given according to the international EEG 10–20 system for electrode placement.

Data were collected using a NIRSport2 optical topography system (manufactured by NIRx Medizintechnik GmbH, Germany). Eight light sources and seven detectors were used, which resulted in a total of 20 channels covering the PFC. The distance of each channel, comprised of one source and one detector, was equal to 3.0 cm. The fiber setup was securely attached to standardized EEG caps with a 10–20 configuration system (see Figure 1). The probe localization was established and applied consistently for each participant using the international 10–20 system positioning; Fz, Cz, and pre‐auricular were measured for each participant. AtlasViewer software was used to identify Montreal Neurological Institute (MNI) coordinates for channels (see Table 2).^41^ Head circumference, ear to ear, and inion to nasion measurements were leveraged for consistency of cap placement.^42^ The data were acquired using the Aurora software (v2014 NIRx Medical Technologies LLC, https://nirx.net/software) at two near‐infrared‐light wavelengths (760 and 850 nm), at a sampling rate of 10.17 Hz.

**TABLE 2:** Functional near‐infrared spectroscopy channels (source and detector) and MNI coordinates determined via AtlasViewer. Channel coordinates are based on the midpoint between the associated source and detector.

Channel	Source	Detector	MNI coordinate (*x*, *y*, *z*)
1	1	1	(−29, 28, 16)
2	1	2	(−11, 32, 32)
3	2	1	(−37, 4, 5)
4	2	3	(−21, 52, 1)
5	3	2	(−19, 55, 34)
6	3	3	(−15, 53, 6)
7	3	4	(−11, 58, 24)
8	4	2	(−9, 48, 56)
9	4	4	(4, 43, 33)
10	4	5	(15, 46, 54)
11	5	3	(−14, 74, 3)
12	5	4	(3, 63, 17)
13	5	6	(14, 73, 1)
14	6	4	(16, 69, 28)
15	6	5	(23, 53, 33)
16	6	6	(25, 58, 10)
17	7	5	(32, 47, 43)
18	7	7	(42, 34, 19)
19	8	6	(34, 63, 0)
20	8	7	(44, 46, 2)

##### fNIRS preprocessing

Data were analyzed using the functional connectivity analysis tool for functional near‐infrared spectroscopy data (fc‐NIRS).^43^ Multiple methods were used to check signal quality; channels were removed if the signal‐to‐noise ratio was less than 2 and if the visual inspections did not detect clear cardiac components in the spectrum (within a range of 1–2 Hz). To be included in the dataset, participants had to have 90% channels passing this threshold, but no participant was excluded for this reason. For motion detection, we used the spline interpolation method and detected motion artifacts by calculating the moving SD with a sliding 2‐s time window. Mean and SD values larger than 5 SD were considered artifacts. To reduce the global noise effect associated with systematic fluctuations, a global signal regression was used by applying a principal component analysis algorithm and filtering out the first component. Data were then band‐pass filtered using a 0.01 Hz high‐pass filter and a 0.1 Hz low‐pass filter (0.01–0.1 Hz), which removed low‐frequency drift (typically below 0.01 Hz) and high‐frequency physiological noise, such as breathing (typically approximately 0.2–0.3 Hz) and cardiac fluctuations (typically approximately 1–2 Hz). Next, data were converted using the modified Beer–Lambert Law using a partial length factor of 6. Correlations between all channels were measured via a 20 by 20 correlation matrix using Pearson method. A Fisher's *Z* transformation was applied to all matrices to standardize the values and to make the data distribution more normal.

### 2.4 Analyses

Of the 60 participants who provided data for at least one assessment, 42 completed all four (fNIRS + IMAS‐R at both time points). Because missingness varied across assessments, analyses used all available data for each comparison (available‐case analysis), resulting in sample sizes that differed slightly across outcomes.

Both fNIRS functional connectivity and IMAS‐R measurements were right‐skewed, so nonparametric approaches were used throughout. Changes in internalizing symptoms between the first and third trimesters were evaluated using the Wilcoxon signed‐rank test. Changes in fNIRS connectivity between the first and third trimesters were assessed with paired *t*‐tests. A two‐sided significance level of 0.05 was used. To control for multiple comparisons, *p*‐values from channel‐level analyses were adjusted using the Benjamini–Hochberg false discovery rate (FDR) procedure.

**FIGURE 2:**
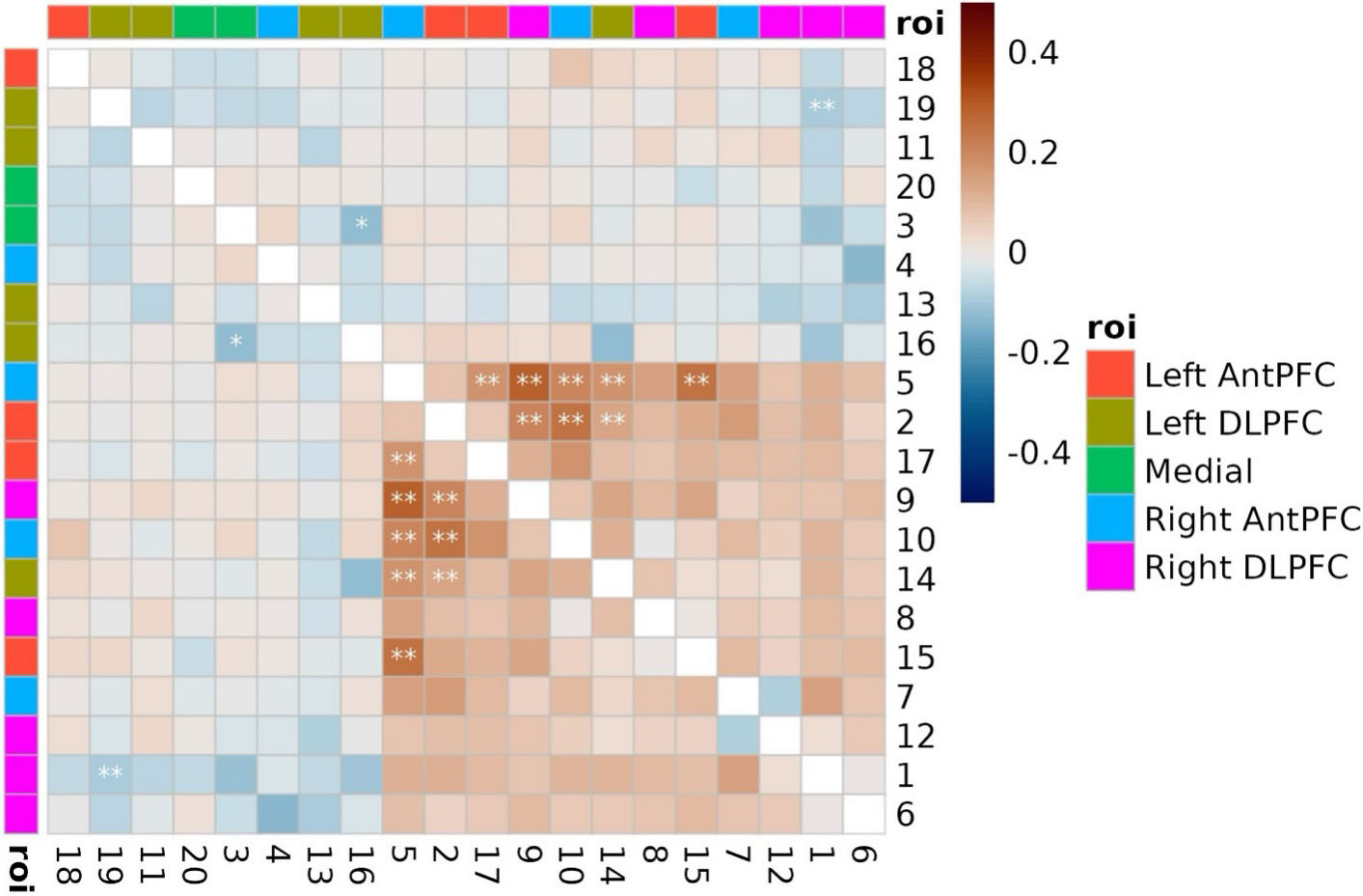
Change in functional connectivity between first and third trimesters of pregnancy. AntPFC, anterior prefrontal cortex; dlPFC, dorsolateral prefrontal cortex; Medial, medial prefrontal cortex; roi, region of interest; legend on right. Channel number is specified on *x* and *y* axes. Shade of color indicates the magnitude of the difference, such that darker colors indicate larger change in connectivity between the two channels. Red colors indicate increased connectivity, and blue colors indicate decreased connectivity. *False discovery rate (FDR) <20%. **FDR <10%.

**FIGURE 3:**
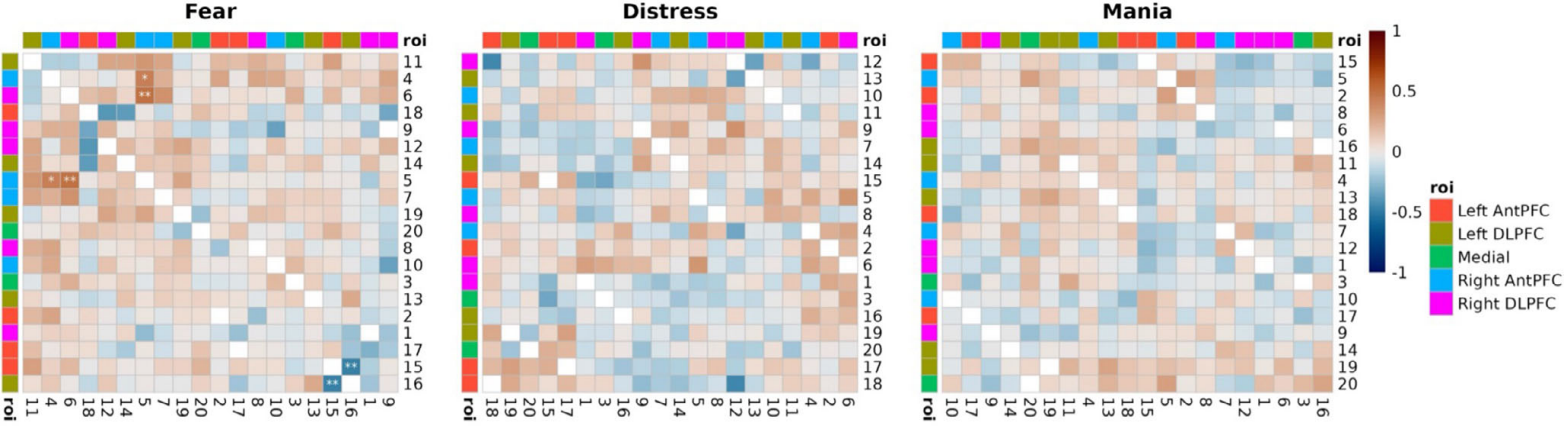
Correlations between connectivity and internalizing symptom domains during the first trimester assessment. AntPFC, anterior prefrontal cortex; dlPFC, dorsolateral prefrontal cortex; Medial, medial prefrontal cortex; roi, region of interest; legend on right. Channel number is specified on *x* and *y* axes. Panel 1 = fear symptoms; Panel 2 = distress symptoms; Panel 3 = OCD/mania symptoms. Channel number is specified on *x* and *y* axes. Shade of color indicates the strength of the correlation, such that darker colors indicate stronger magnitude of correlation between connectivity and symptom domain. Red colors indicate positive correlations, and blue colors indicate negative correlations. *False discovery rate (FDR) <20%. **FDR <10%.

**FIGURE 4:**
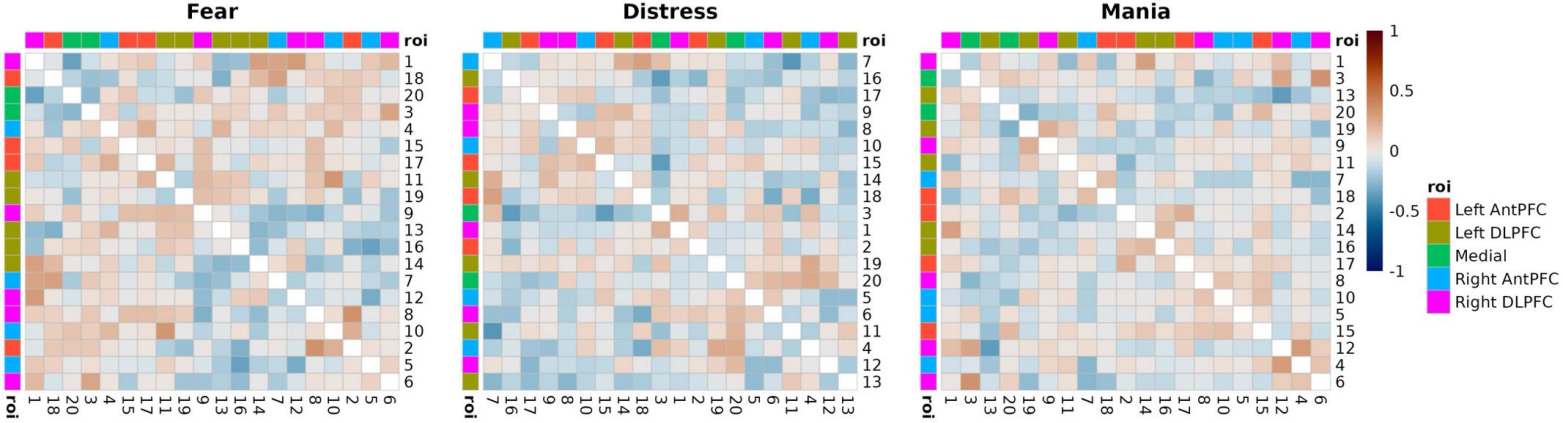
Correlations between connectivity and internalizing symptom domains during the third trimester assessment. AntPFC, anterior prefrontal cortex; dlPFC, dorsolateral prefrontal cortex; Medial, medial prefrontal cortex; roi, region of interest; legend on right. Channel number is specified on *x* and *y* axes. Panel 1 = fear symptoms; Panel 2 = distress symptoms; Panel 3 = OCD/mania symptoms. Channel number is specified on *x* and *y* axes. Shade of color indicates the strength of the correlation, such that darker colors indicate stronger magnitude of correlation between connectivity and symptom domain. Red colors indicate positive correlations, and blue colors indicate negative correlations. None of the associations between connectivity and internalizing symptom domains at the third trimester time point were significant after adjustment for multiple testing.

**FIGURE 5:**
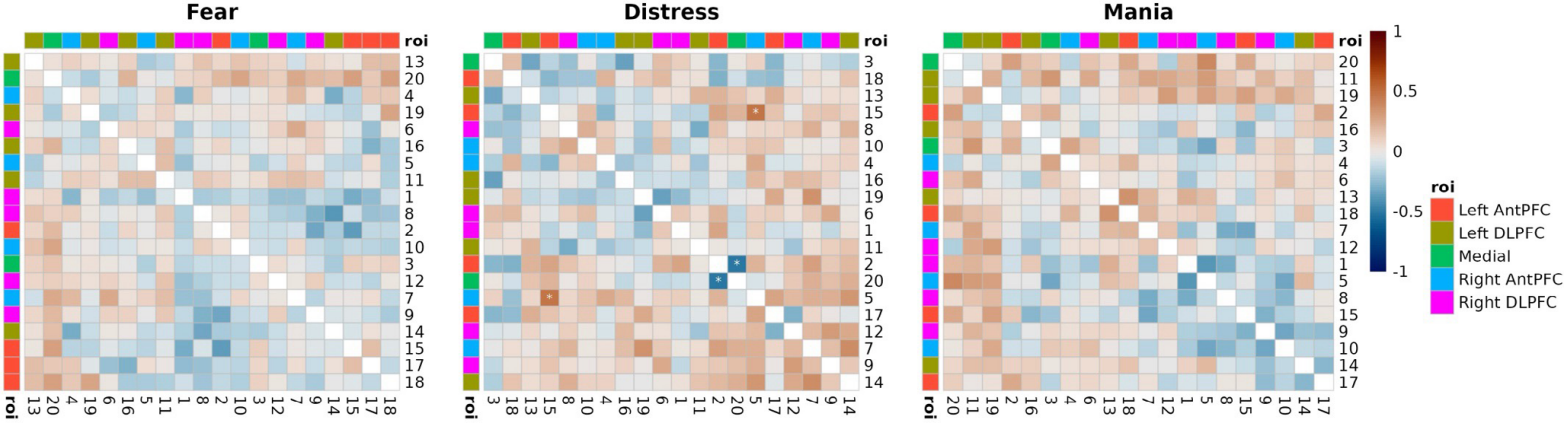
Change in connectivity as related to change in internalizing symptom domains between first and third trimesters of pregnancy. AntPFC, anterior prefrontal cortex; dlPFC, dorsolateral prefrontal cortex; Medial, medial prefrontal cortex; roi, region of interest; legend on right. Channel number is specified on *x* and *y* axes. Panel 1 = fear symptoms; Panel 2 = distress symptoms; Panel 3 = OCD/mania symptoms. Channel number is specified on *x* and *y* axes. Shade of color indicates the strength of the correlation, such that darker colors indicate stronger magnitude of correlation between the changes. Red colors indicate positive correlations, and blue colors indicate negative correlations. *False discovery rate (FDR) <20%. **FDR <10%.

Associations between internalizing symptom domains (distress, fear, and OCD/mania) and functional connectivity were assessed using Spearman rank correlation, with significance tested using the *t*‐approximation. To evaluate whether changes in internalizing symptom domains were associated with changes in functional connectivity, individual‐level differences (third trimester minus first trimester) were calculated for both internalizing symptoms and connectivity measures. These differences were then rank‐normalized (ranks transformed to a standard normal distribution with mean 0 and variance 1) before fitting linear regression models. Models were fit both with and without adjustment for baseline internalizing symptom values (distress, fear, and OCD/mania domains). Finally, associations between baseline internalizing symptom domains and subsequent changes in functional connectivity were examined using the same rank‐normalization and linear regression approach. For the heatmaps (Figures 2, 3, 4, 5), hierarchical clustering was used to order the channels, so channels with similar patterns appear next to one another. Note that the order of the channels changes from figure to figure as a result, since it is data dependent.

## 3 RESULTS

### 3.1 Change in internalizing symptoms (IMAS‐R)

At the first trimester, the mean distress score was 26.68 (SD = 21.90, range: 0–93); the mean fear score was 9.12 (SD = 10.50, range: 0–44); and the mean OCD/mania score was 3.73 (SD = 6.25, range: 0–33). At the third trimester, the mean distress score was 17.65 (SD = 16.58, range: 0–82); the mean fear score was 5.22 (SD = 6.93, range: 0–38); and the mean OCD/mania score was 1.73 (SD = 3.16, range: 0–14). For visualization, see Supporting Information S1: Figure 1.1. We first examined changes in the three domains of internalizing symptoms: distress, fear, and OCD/mania between the first and third trimesters using the Wilcoxon signed‐rank test among 49 participants who completed both first and third trimester IMAS‐Rs. There was a significant decrease in distress from first to third trimester (from a median of 21 to 13, *p* < .001) and in fear symptoms (change in median from 6 to 2, *p* = .007). Overall, OCD/mania symptoms were uncommon, with 43 participants receiving a score of 0 in the first trimester and 41 participants receiving a score of 0 in the third trimester (no significant change, *p* = .7). Given the lack of variability in the OCD/mania domain, we did not proceed with further cross‐sectional or longitudinal analyses with this domain.

### 3.2 Change in connectivity

Mean levels of connectivity are displayed in Supporting Information S1: Figure 1.2. We examined changes in the functional connectivity between the first and the third trimester using paired *t*‐tests among 42 participants who completed first and third trimester fNIRS. We analyzed changes at the channel level. At the channel level, using a 10% FDR threshold, we observed significant increases in connectivity between the following channel pairs: 2 × 9, 2 × 10, 2 × 14, 5 × 9, 5 × 10, 5 × 14, 5 × 15, and 5 × 17 (see Supporting Information S1: Figure 2.1). All eight increases involved one of the two left‐hemisphere medial PFC (channel 2 or 5) connecting to either contralateral or medial channels. These primarily correspond to changes in BA 8, 9, and 10. These changes overall reflect a medial anchoring, specifically a strengthening of medial‐frontal connectivity and integration of left medial regions with other right prefrontal regions. The only channel combination that showed a decrease was 1 × 19, corresponding to reduced interhemispheric connectivity between left inferolateral (corresponding to BA 46) and right middle frontal areas (corresponding to BA 46/10). Figure 2 conveys the results, and channels were grouped by region for ease of interpretation (regions are determined based on MNI coordinates).

### 3.3 Correlation between IMAS‐R and connectivity

We first examined correlations between IMAS‐R and PFC connectivity cross‐sectionally within each trimester and then examined the relations of longitudinal changes in IMAS‐R to connectivity. Because levels of OCD/mania symptoms were low overall with little variability, we did not further explore relations of OCD/mania to connectivity patterns. In the first trimester, higher levels of fear were associated with two significant (FDR <10%) functional connectivity patterns: mothers with higher symptoms of fear exhibited *lower* within‐region connectivity in right medial PFC in channel pair 15 × 16 (*r* = −0.48), and *higher* within‐region connectivity in left medial PFC in channel pair 5 × 6 (*r* = 0.49). These primarily corresponded to BA 8, 9, and 10. A third pair, 4 × 5, connecting left dorsolateral to left medial nodes (*r* = 0.43), reached borderline significance (17% FDR). Figure 3 conveys the findings from the first trimester, and Supporting Information S1: Figure 2.2 conveys scatterplots.

None of the associations between connectivity and internalizing symptom domains at the third trimester time point were significant after adjustment for multiple testing (see Figure 4).

Finally, we investigated whether changes in IMAS‐R dimensions between the first and third trimester related to changes in connectivity. No associations were seen at the 10% FDR level of significance, but changes in distress were associated with multiple changes in connectivity at the less stringent 20% FDR threshold. Decreases in distress from first to third trimester were associated with an increase in interhemispheric connectivity between channels 2 and 20 (*r* = 0.50), representing a left medial to right lateral prefrontal coupling, and with a decrease in interhemispheric connectivity in the 5 × 15 channel pair, representing a weakened coupling between homologous bilateral medial frontal regions (*r* = −0.47). See Figure 5 for a graphical depiction of the findings and Supporting Information S1: Figure 2.2 for scatterplots.

## 4 DISCUSSION

The present study sought to fill important gaps in the perinatal literature, including expanding observational longitudinal work, measuring psychopathology using a dimensional structured interview (IMAS‐R), and associating neuroconnectivity and psychopathology measures starting in the first trimester. We add to the existing literature by providing a longitudinal assessment of prefrontal functional connectivity with fNIRS during pregnancy, together with dimensional internalizing symptoms. The findings from the present study indicated that distress and fear domains of internalizing symptoms decreased from the first to third trimester, and OCD/mania symptoms were low on average and had limited variability at both time points. Neuroconnectivity changes from the first to third trimester were demonstrated bilaterally, with increases anchored on left medial frontal nodes and extending to medial, anterior, and lateral prefrontal regions. Cross‐sectional associations between internalizing symptom domains and neuroconnectivity were primarily between fear and within‐region coupling in the bilateral medial frontal cortex, but with opposing patterns in the two hemispheres. Longitudinal analyses indicated that decreases in distress were associated with stronger connectivity of left medial frontal regions to right lateral prefrontal areas, but these did not reach statistical significance at the most stringent threshold. Although relatively few associations survived correction for multiple comparisons, the patterns were consistent with our hypotheses, and effect sizes were quite strong.

Decreases in distress and fear symptoms between first and third trimesters aligned with our hypotheses. This finding also aligns with prior research indicating that generalized anxiety and pregnancy‐related anxiety decrease between first and third trimesters.^44^, ^45^, ^46^ Others have conceptualized this as a normal and adaptive process, suggesting that concern for the developing fetus' health can result in compliance with medical advice.^47^ Moreover, fear of birth concerns across trimesters are particularly relevant as parents learn about birthing processes and make birthing plans, with prior work demonstrating that fear of birth decreased in prevalence from mid‐pregnancy to late pregnancy.^48^ Anxiety may be higher during the first trimester as the first ultrasound occurs, and women may grow more familiar with the ultrasound process as the pregnancy progresses. Moreover, women may experience more physical discomfort during the first trimester. In addition to examining changes in anxiety, other prior literature has examined changes in depressive symptoms between pregnancy and the postpartum period,^6^, ^49^ and the current work adds much‐needed information about within‐pregnancy change. Prior work has investigated obsessive‐compulsive spectrum symptoms during pregnancy and postpartum, but more work is needed.^50^ A study that measured symptoms across obsessive‐compulsive and related disorders found that few participants (6.2%) endorsed clinically significant symptoms during pregnancy, but at least twice as many (14%) endorsed clinically significant obsessive‐compulsive symptoms during the postpartum period.^51^ As such, our findings of limited obsessive‐compulsive symptoms during pregnancy align with prior work and extend these findings across multiple time points during pregnancy. Future work in the postpartum period will be necessary to replicate findings of elevated OCD symptoms during the postpartum period. Moreover, measurement of symptoms across the internalizing spectrum is critical, as dimensional measurement allows for comorbid symptoms without rigid adherence to diagnostic categories.

Our second hypothesis was that prefrontal resting‐state functional connectivity would increase from the first to third trimester, and this hypothesis was supported by a subset of channels in the medial frontal and dorsal frontal areas. All increases involved two adjacent channels in left dorsomedial areas connecting to right hemisphere including contralateral medial, dorsolateral, and anterior regions. Stated differently, we saw a strengthening of interhemispheric integration anchored on the left medial PFC, extending bilaterally to right medial, anterior, and dorsolateral prefrontal regions. This aligns with prior literature utilizing resting‐state fMRI, wherein enhanced global and local network efficiency in prefrontal areas was observed in primiparous women compared to nulliparous women, and increased temporal coherence of prefrontal areas was observed between pre‐pregnancy and the postpartum period in cross‐sectional designs.^12^, ^29^ Moreover, other work examining changes between pregnancy and postpartum using EEG coherence found that left intrahemispheric coherence was increased in pregnancy relative to postpartum.^30^ Here we add to the literature by reporting functional changes during pregnancy in a longitudinal sample.

The increases in connectivity that we observed in this study may partially reflect increased mentalizing, introspection, and attention to internal states during pregnancy, which is one of the functions of medial prefrontal areas.^30^ Prior research has demonstrated that within prefrontal areas specifically, the medial prefrontal areas serve as main hubs of the DMN, and they are involved in self‐referential thinking and evaluating an infant's emotional cues. Theory of mind networks are also often attributed to the medial PFC, among other areas in the brain.^52^ At the same time, lateral prefrontal regions are considered to be involved in more effortful emotion regulation, interacting with medial regions during internally directed thought.^20^ Increasing coupling within and between medial frontal nodes bilaterally (which extended into anterior and lateral prefrontal regions) could represent an adaptive response as one prepares for parenthood. Parents must be able to reflect upon their internal states to assist their infant in navigating early life experiences. Thus, preparing for this as pregnancy progresses is an expected process. Previous research has framed emotion regulation as a core developmental process in parenthood,^53^ and other empirical papers have noted structural changes during pregnancy versus postpartum in areas that also subserve theory of mind.^11^ Each of these findings may support the notion that greater attention to internal states and the behavioral responses to them is likely a process that increases during pregnancy as one prepares for the arrival of the infant. These networks also serve other functions, including working memory,^52^ cognitive control,^54^ and language organization,^55^ which should be explored in future work to determine the role of multiple mechanisms underlying these changes in outcomes during pregnancy.

Our third aim was to examine the relations between internalizing symptom domains and prefrontal connectivity cross‐sectionally and longitudinally between first and third trimesters. Here we saw more localized and lateralized relations. At the first trimester time point, higher fear symptoms were associated with hemispherically asymmetric patterns of within‐region coupling in homologous bilateral medial frontal regions. Fear was related to stronger coupling within left medial prefrontal areas, but weaker coupling in right medial prefrontal areas. Thus, while overall pregnancy‐related effects seemed to indicate broader reorganization of prefrontal areas, fear‐related differences were specific to asymmetric modulation within bilateral medial superior frontal areas. Broadly, our results are consistent with prior task‐based fNIRS research utilizing experimental fear‐relevant stimuli which found that increased prefrontal activation was associated with stress, anxiety, cortisol, and testosterone among pregnant women compared to controls.^25^ The medial PFC is one of the most consistently implicated areas when healthy individuals are induced with anxiety.^56^ The asymmetric pattern we observed, stronger left‐medial coupling and weaker right‐medial coupling with higher fear, is consistent with broader frameworks of left‐versus‐right prefrontal asymmetry in stress regulation,^57^, ^58^ which we extend here to the medial frontal cortex. Left medial PFC has been implicated in self‐focused and internally directed processing, such as rumination or worry.^59^ Sandoval et al.^30^ reported correlations between postpartum depression scores and higher left‐hemisphere frontopolar‐parietal coherence, suggesting that left‐hemisphere medial‐frontal engagement may track internalizing symptoms across the peripartum period. Conversely, reduced right medial‐frontal coherence may reflect attenuated engagement of right‐hemisphere networks implicated in emotion regulation and inhibition.^58^ Right frontal areas have also been suggested to play a role in attending to nonverbal, emotionally salient cues from the infant.^57^ Consistent with this interpretation, a recent task‐based fNIRS study linked increases in right prefrontal area activation to more sensitive parenting.^26^ Overall, our results add to the emerging literature on neural changes during pregnancy and suggest that pregnancy is accompanied by dynamic changes in prefrontal resting state functional connectivity, which are in turn tied to internalizing symptoms.

When examining the longitudinal associations between connectivity and internalizing symptom domains, decreases in distress from first to third trimester were associated with increased connectivity between left medial PFC and right lateral PFC, alongside weakened connectivity between homologous bilateral medial frontal nodes. This spatial pattern suggests that as distress decreased, the coupling shifted from intrahemispheric medial integration toward a broader medial to lateral integration across hemispheres. These results are also informative when considered in relation to findings surrounding fear. We observed within‐hemisphere coupling with symptoms of fear, but longitudinal findings around distress reflect a more cross‐hemisphere medial to lateral integration pattern. Taken together, our results highlight that the changes in the brain are functionally linked to changes in maternal internalizing symptoms in this sample. The relation between internalizing symptoms and the specific pattern of neurological changes is multifaceted, as opposed to simple unidirectional associations, underscoring the importance of longitudinal work. Diverging relations are also consistent with the idea that neural changes in pregnancy are not uniformly directional.^12^, ^14^ Finally, medial–lateral prefrontal connectivity supports emotion regulation and is frequently implicated in studies involving depression and anxiety,^32^, ^33^, ^34^ possibly suggesting that pregnancy‐related increases may index a regulatory reorganization rather than a maternal‐specific process. Taken together, the present study contributes to the literature by pinpointing different profiles of change in the brain as a function of pregnancy, as well as elucidating relations to changing internalizing symptoms. A multitude of factors at multiple levels of analyses, including biological, environmental, and experiential, likely predict these changes we observed. For example, it is well‐established that there are significant changes in the cellular and molecular mechanisms, such as neurogenesis.^60^ Other possible mechanisms include changes in gestational hormonal processes which show extreme fluctuations during pregnancy into postpartum periods.^15^, ^60^ Last but not least, psychosocial influences and changes in the environment are likely to contribute to the changes observed, as motherhood tends to bring a diverse array of additional mental and physical tasks upon the individual.^61^

### 4.1 Strengths and limitations

There are several strengths of the present study that ought to be considered alongside its limitations. We utilized fNIRS, a portable, safe, and non‐invasive method of measuring connectivity. fNIRS enables higher temporal resolution and denser, repeated sampling than fMRI; however, relative to fMRI, fNIRS has lower spatial resolution. In addition, resting‐state functional connectivity reflects the temporal correlation of spontaneous hemodynamic fluctuations, but not the specific direction of information flow between nodes. Our fNIRS cap primarily focused on prefrontal areas, given prior literature to support further investigation of connectivity in these areas.^11^, ^22^, ^35^, ^38^ We could not and therefore did not examine connectivity in subcortical regions, which would be a beneficial direction for future research but cannot be done using fNIRS.

A key strength is that the study assessed two within‐pregnancy time points using fNIRS. Existing human work has largely relied on pre‐ to post‐pregnancy comparisons^11^ or pregnant/postpartum‐versus‐nulliparous contrasts,^13^ capturing the transition only at its endpoints; even recent within‐gestation sampling has been confined to structural MRI in single individuals.^14^

In the present work, we operationalized internalizing symptoms in a dimensional manner, which aligns with the hierarchical taxonomy of psychopathology, rather than categorical diagnoses. Given that diagnoses are known to covary, and symptom severity is expected to fluctuate during periods characterized by biopsychosocial change (like pregnancy), this conceptualization better fits with the extant psychopathology literature. Moreover, considering prior work that has demonstrated elevations in anxiety, obsessive‐compulsive, posttraumatic stress, and mania symptoms during the perinatal and postpartum periods,^2^, ^62^, ^63^, ^64^ it is critical that a broad and robust assessment of these symptoms be utilized to fully characterize symptom change throughout pregnancy. The IMAS‐R is a structured interview that was administered by trained and formally tested assessors, and it offered three broad subdomains—distress, fear, and OCD/mania—that cut across diagnostic categories. This study was conducted with a community sample of women with prenatal care, so we anticipated and collected a sample with a range of symptom severity. This further underscores the importance of the dimensional approach, given that no inclusion or exclusion criteria were applied related to diagnoses, symptoms, or engagement in mental healthcare. Although OCD/mania means were low during pregnancy, future work will characterize postpartum levels in this sample, as well as how changes in this subdomain relate to changes in the brain. Prior work has indicated elevated OCD symptoms during the postpartum period,^63^, ^64^ so longitudinal work examining predictors of elevations will be particularly important. Critically, we did not assess externalizing symptoms or psychotic‐like symptoms in this study. Future work should characterize these symptoms, among others, to ensure the full range of psychopathology is assessed.

Despite attempts to recruit all eligible pregnant individuals in prenatal care who were interested in participating, our sample was demographically homogenous. Future work should seek to include individuals who are not involved in prenatal care to ensure that samples are not biased toward more well‐resourced individuals. The sample was relatively small; although sufficient to identify large effects, the study was underpowered to reliably detect small‐to‐moderate effects after correction for multiple comparisons. Attrition was expected, such that some participants completed parts of the assessments (e.g., completed interview but opted out of fNIRS), and missing data may have impacted our findings. To be included in each of the analyses described herein, participants needed to opt in for completion of IMAS‐R and fNIRS at both pregnancy time points. While 42 individuals completed each of these four assessments, others did not (three completed only IMAS‐R at both time points, one did not complete first trimester fNIRS, two did not complete third trimester fNIRS, 11 completed only first trimester assessments, and one completed only third trimester assessments). As such, our findings are limited to those who were most engaged in the study across time points. Given that engagement in all fNIRS was opt‐in only, and participants were free to decline to participate in any part of the study at any point, attrition was expected; nevertheless, attrition undermines confidence in the generalizability and representativeness of our findings. Finally, the present study did not examine the potential effect of parity on our findings. Data for this variable were collected after the present analyses in the postpartum period due to author oversight in measuring this variable prospectively. Within our sample, 33.3% of participants were primiparous, 46.7% were multiparous, and 20% were missing this information. Additionally, there are many factors that were not examined in our study, including hormonal changes throughout pregnancy, which will be important future research directions.

### 4.2 Future directions

This study makes novel contributions to the literature by examining longitudinal associations during pregnancy between fNIRS‐derived neuroconnectivity and dimensional internalizing symptoms; however, important questions still remain. Most notably, the present work captured two time points during pregnancy, despite this being a period that lasts for approximately 9 months. There is much to be gained from denser, more granular data that captures week‐to‐week or within‐day change. In addition, integrating data that assesses hormonal fluctuations throughout pregnancy is important to identify how these changes associate with changes in connectivity and internalizing symptoms. We did not pinpoint relations to parent or infant outcomes. The parent study from which these data were taken will continue to follow up with participants and their infants throughout the postpartum period. Examining the impact of these changes on the development of the infant will be an essential next step. Further work in replicating or extending these findings in a larger and more racially, ethnically, and socioeconomically diverse sample will be critical. This sample included participants who were connected with prenatal care, and the sample was demographically homogenous. As such, the findings may be more pronounced in a sample with fewer healthcare resources. Moreover, examining these questions in a sample with clinical diagnoses or those in active treatment may be helpful comparisons or extensions of the present work. In addition, the fNIRS session included a resting‐state task, which offers advantages when associated with symptoms. Beyond resting‐state data, examining functional task connectivity would be a useful extension of this work, as differences in processing of emotional stimuli have been observed in the context of pregnancy.^25^, ^26^ Finally, identifying malleable and targetable processes that can explain these changes in connectivity and symptoms is a crucial next step toward future prevention and intervention efforts to minimize adverse outcomes.

### 4.3 Conclusions

Pregnancy is a period characterized by biopsychosocial change. The present study examined resting‐state prefrontal connectivity measured with fNIRS in a sample of pregnant individuals assessed during the first and third trimesters of pregnancy and sought to identify the associations to changes in internalizing symptom domains. The findings identified dorsolateral and medial prefrontal regions as key areas of change in resting‐state functional connectivity during pregnancy, with alterations in prefrontal connectivity being associated with distress internalizing symptoms measured using a well‐validated structured interview, the IMAS‐R. These results highlight the promise of fNIRS and longitudinal neuroimaging as means to identify risk during this critical developmental period. Future work should examine these questions in larger samples to replicate and extend these findings. Because pregnancy elevates risk for psychopathology, neuroimaging measures such as fNIRS can serve as non‐invasive markers of risk that could be utilized for prevention and intervention efforts, ultimately improving outcomes for parents and family units alike.

## AUTHOR CONTRIBUTIONS

**Emily B. K. Thomas:** Conceptualization; methodology; investigation; funding acquisition; writing – original draft; writing – review and editing; supervision; resources; project administration. **Micah A. Williams:** Data curation; writing – original draft; writing – review and editing; methodology. **Patrick Breheny:** Visualization; formal analysis; writing – original draft; writing – review and editing. **Abigél Miskolczi:** Writing – review and editing; methodology. **Ö. Ece Demir‐Lira:** Conceptualization; funding acquisition; writing – original draft; writing – review and editing; resources; methodology; supervision; investigation; project administration.

## CONFLICTS OF INTEREST

The authors have no conflicts of interest to disclose.

## Supporting information


**Data S1.** Supporting Information.

## Data Availability

The research data are not shared due to ethical restrictions.
