# Supplementary material for: Examining dynamic changes in prefrontal connectivity and internalizing symptoms during the first and third trimesters of pregnancy
Source: J Neuroendocrinol. 2026 Sep 4;38(9):e70252. doi: 10.1111/jne.70252 (PMC13543730; doi:10.1111/jne.70252)

Supplementary Results

Examining dynamic changes in prefrontal connectivity and internalizing symptoms during the first and third trimesters of pregnancy

Emily B. K. Thomas   Micah A. Williams   Patrick Breheny
Abigél Miskolczi   Ece Demir-Lira

Table of contents

1 Univariate graphical summaries **1**
1.1 IMAS . . . 1
1.2 fNIRS . . . 2

2 Visualization of significant results **2**
2.1 Changes in functional connectivity . . . 2
2.2 Association between IMAS and functional connectivity . . . 3

1 Univariate graphical summaries

1.1 IMAS

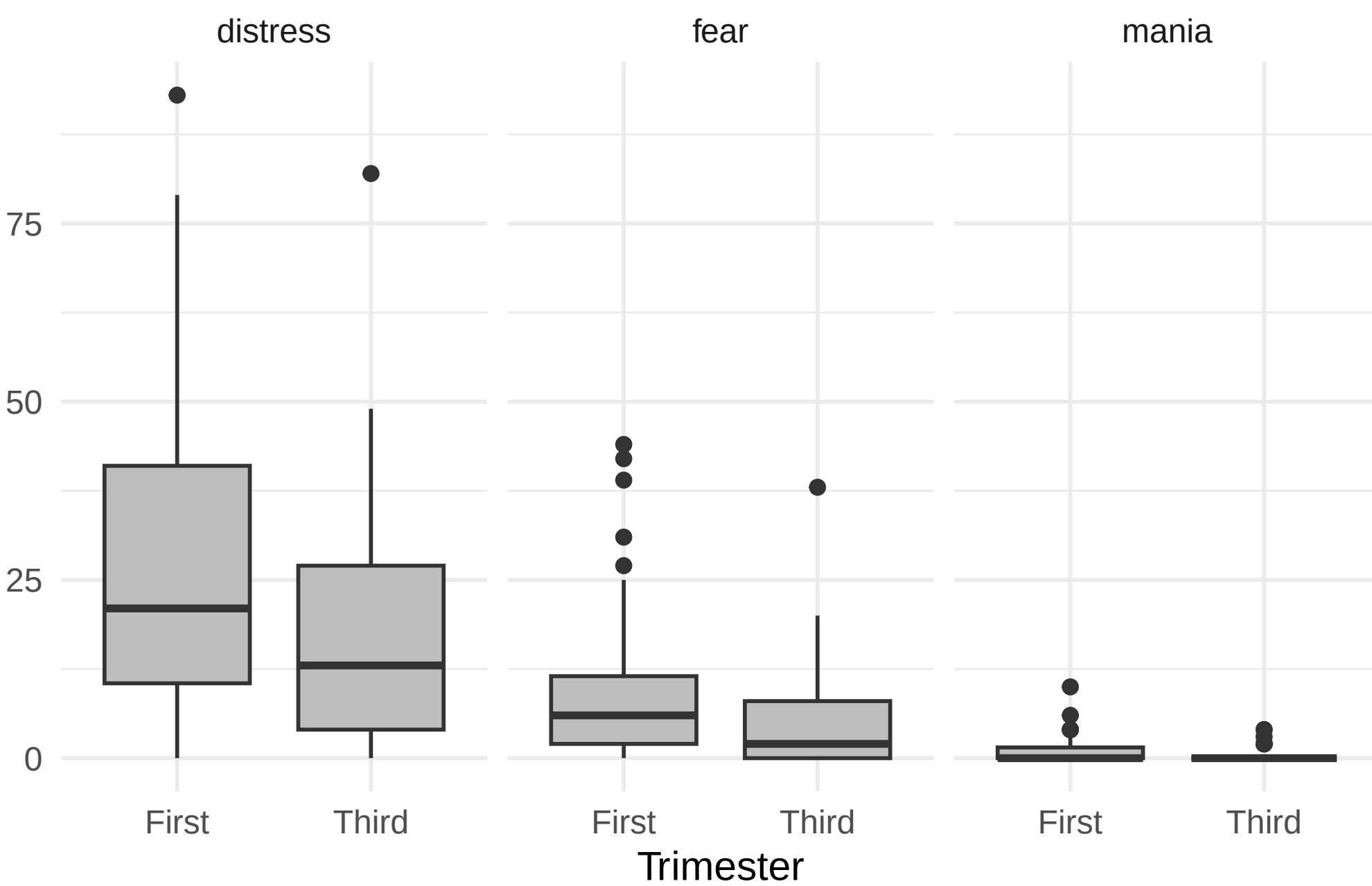

1.2 fNIRS

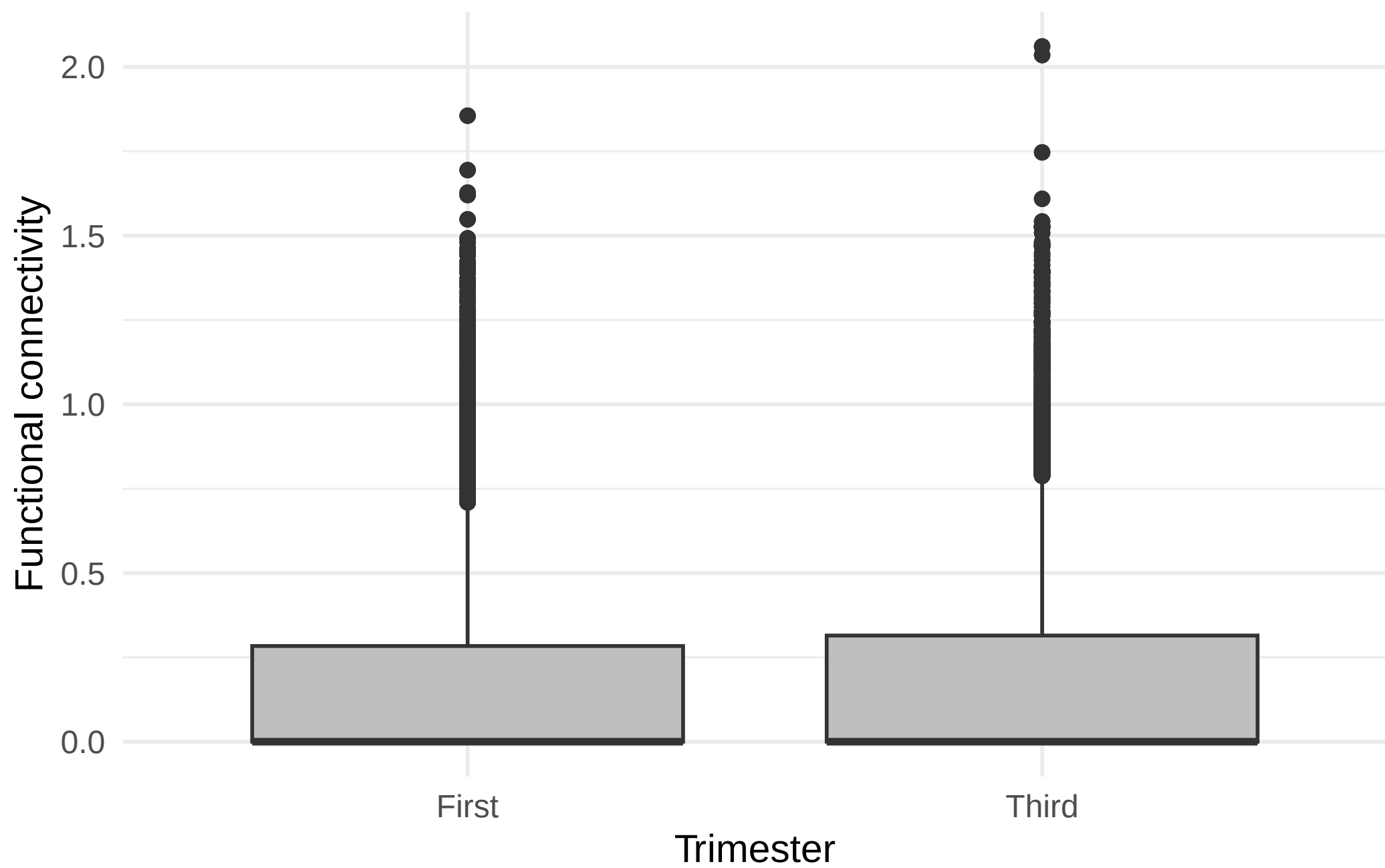

2 Visualization of significant results

2.1 Changes in functional connectivity

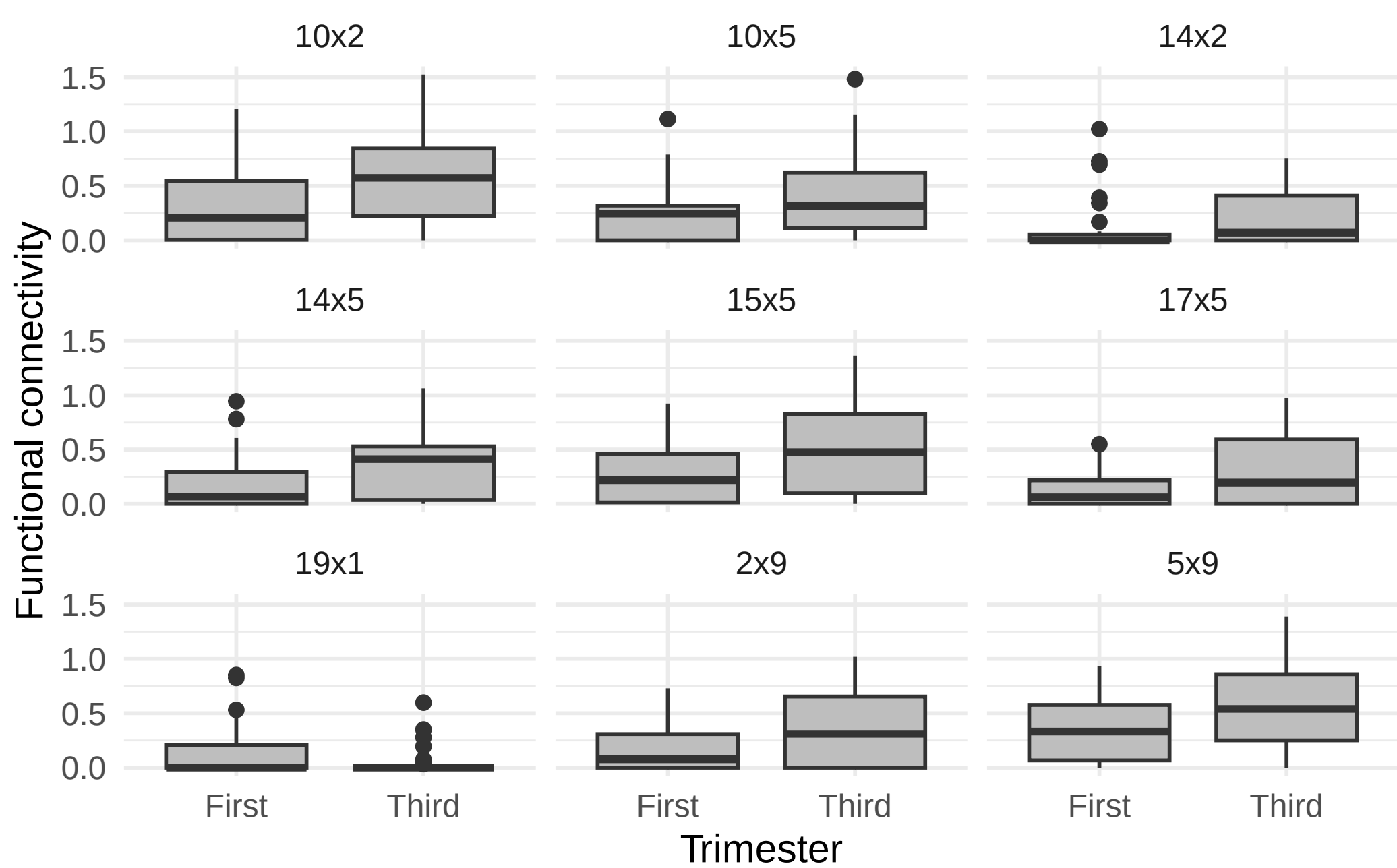

2.2 Association between IMAS and functional connectivity

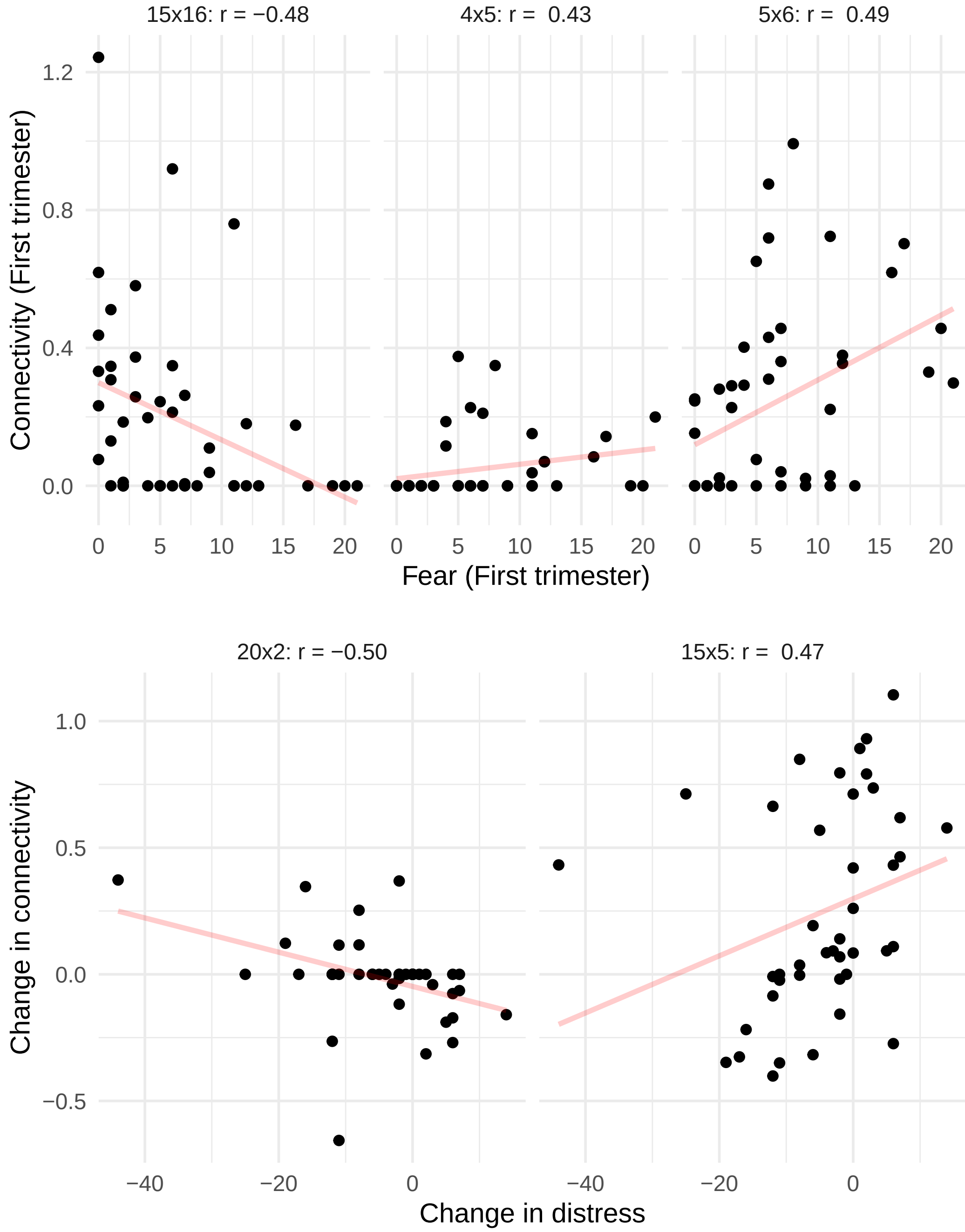

Supplement: Supplementary file 1 [file JNE-38-e70252-s001.pdf]
